# DJ-1 Null Dopaminergic Neuronal Cells Exhibit Defects in Mitochondrial Function and Structure: Involvement of Mitochondrial Complex I Assembly

**DOI:** 10.1371/journal.pone.0032629

**Published:** 2012-03-05

**Authors:** Jun Young Heo, Ji Hoon Park, Soung Jung Kim, Kang Sik Seo, Jeong Su Han, Sang Hee Lee, Jin Man Kim, Jong Il Park, Seung Kiel Park, Kyu Lim, Byung Doo Hwang, Minho Shong, Gi Ryang Kweon

**Affiliations:** 1 Department of Biochemistry, Chungnam National University School of Medicine, Daejeon, Korea; 2 Research Center for Endocrine and Metabolic Diseases, Department of Internal Medicine, Chungnam National University School of Medicine, Daejeon, Korea; 3 Department of Pathology, Chungnam National University School of Medicine, Daejeon, Korea; 4 Research Institute for Medical Science, Chungnam National University School of Medicine, Daejeon, Korea; 5 Infection Signaling Network Research Center, Chungnam National University School of Medicine, Daejeon, Korea; University of Pittsburgh, United States of America

## Abstract

DJ-1 is a Parkinson's disease-associated gene whose protein product has a protective role in cellular homeostasis by removing cytosolic reactive oxygen species and maintaining mitochondrial function. However, it is not clear how DJ-1 regulates mitochondrial function and why mitochondrial dysfunction is induced by DJ-1 deficiency. In a previous study we showed that DJ-1 null dopaminergic neuronal cells exhibit defective mitochondrial respiratory chain complex I activity. In the present article we investigated the role of DJ-1 in complex I formation by using blue native-polyacrylamide gel electrophoresis and 2-dimensional gel analysis to assess native complex status. On the basis of these experiments, we concluded that DJ-1 null cells have a defect in the assembly of complex I. Concomitant with abnormal complex I formation, DJ-1 null cells show defective supercomplex formation. It is known that aberrant formation of the supercomplex impairs the flow of electrons through the channels between respiratory chain complexes, resulting in mitochondrial dysfunction. We took two approaches to study these mitochondrial defects. The first approach assessed the structural defect by using both confocal microscopy with MitoTracker staining and electron microscopy. The second approach assessed the functional defect by measuring ATP production, O_2_ consumption, and mitochondrial membrane potential. Finally, we showed that the assembly defect as well as the structural and functional abnormalities in DJ-1 null cells could be reversed by adenovirus-mediated overexpression of DJ-1, demonstrating the specificity of DJ-1 on these mitochondrial properties. These mitochondrial defects induced by DJ-1mutation may be a pathological mechanism for the degeneration of dopaminergic neurons in Parkinson's disease.

## Introduction

Studies of Parkinson's disease (PD), the second most common neurodegenerative disease after Alzheimer's, have focused on mitochondrial respiratory chain complex I since the discovery in 1990 that complex I activity is reduced in the substantia nigra of PD patients [1]. Impairment of the respiratory chain disrupts electron transfer and generates oxidative stress, resulting in mitochondrial dysfunction that can lead to cell death through apoptosis. Furthermore, mitochondrial dynamics (e.g., fission, fusion, motility, mitophagy, etc.) are important for the maintenance of mitochondrial functions in neurons [2], [3], and there is growing recognition that abnormal mitochondrial dynamics may also contribute to the pathogenesis of PD. Ensuring mitochondrial quality and appropriate energy supplies are essential for normal neuronal activities [4]. Consequently, maintenance of mitochondrial function is one of the most important targets for preventing and treating neurodegenerative diseases, and it is therefore important to understand the factors that regulate both the respiratory chain and mitochondrial dynamics.

The interrelationship between complex I impairment and mitochondrial morphologic changes in PD development has been studied in both toxin-induced and genetic models of PD [5]. Some of the most common environmental causative factors of PD are known to target complex I. Before the onset of chemically induced dopaminergic neuronal cell death, neurotoxins (e.g., 6-hydroxydopamine; 6-OHDA) and pesticides (e.g., rotenone) that impact mitochondria increase the levels of mitochondrially generated reactive oxygen species (ROS), resulting in altered mitochondrial dynamics and subsequent fragmentation [6]. PD-related gene mutations or gene deletions show mitochondrial respiratory chain defects and induce fission-like morphologic changes. For example, Pink1 and Parkin deficiencies were shown to decrease mitochondrial respiratory chain activity and impair mitochondrial fusion in flies [7] and promote mitochondrial fragmentation in mammals [8], and DJ-1-deficient PD patients have smaller mitochondria [9]. Pink1 and Parkin may function in the regulation of mitochondrial dynamics for mitochondrial quality control, which involves ridding the cell of damaged mitochondria via induction of mitophagy. However, the mitochondrial function of DJ-1 needs to be further evaluated.

Mutation of DJ-1, which causes a rare, autosomal recessive form of PD, is postulated to result in the breakdown of antioxidant defenses in various cellular compartments. DJ-1 can prevent oxidative damage in the cytosol by acting in a manner similar to glutathione peroxidase [10]. Mitochondrial DJ-1 has a role in balancing mitochondrial dynamics, and DJ-1 deficiency can cause mitochondrial fragmentation [9]. It is thought that a defect in complex I is the main cause of mitochondrial fragmentation in DJ-1 deficiency, but the mechanism of this pathologic change is not clear.

Formation of complex I depend on three steps: translation of mitochondrial subunits in both the mitochondrion and cytoplasm, importation of the cytoplasmic subunits, and subsequent assembly of the subunits into complex I. To investigate how DJ-1 mutations affect the integrity of mitochondrial complexes, we performed biochemical experiments by using blue native-polyacrylamide gel electrophoresis (BN-PAGE) and functional assays. Our goal in this study was to determine the role of DJ-1 in complex I formation and how it affects mitochondrial function in PD-related dopaminergic neuronal cells. Based on our results, we propose that DJ-1 mutation contributes to the pathogenesis of PD through impairment of the complex I assembly pathway.

## Results

### Mitochondrial complex I is not properly assembled in DJ-1 null cells

According to studies of human PD, mutation of DJ-1 results in perturbed mitochondrial dynamics [9], [11]. We hypothesized that mitochondrial pathologic changes caused by DJ-1 mutation result from abnormalities of respiratory complex subunits. To identify the function of DJ-1 in mitochondria, we examined mouse DJ-1 null dopaminergic neuronal cells, which reflect human PD pathogenesis in a murine cell system. First, we compared the expression levels of mitochondrial complex subunits in SN4741 (‘wild-type’) and DJ-1 null cells (Figure S1). No differences were observed in mitochondrial subunit expression between these cell lines, with the exception of COX4, which is a subunit of mitochondrial complex IV. The absence of a general defect in expression of mitochondrial complex subunits indicates that DJ-1 is not involved in the translation of mitochondrial complex subunits; however, the increased level of the mitochondrial respiratory chain subunit COX4 in DJ-1 null cells implies an increase in damaged mitochondria [12].

**Figure 1:**
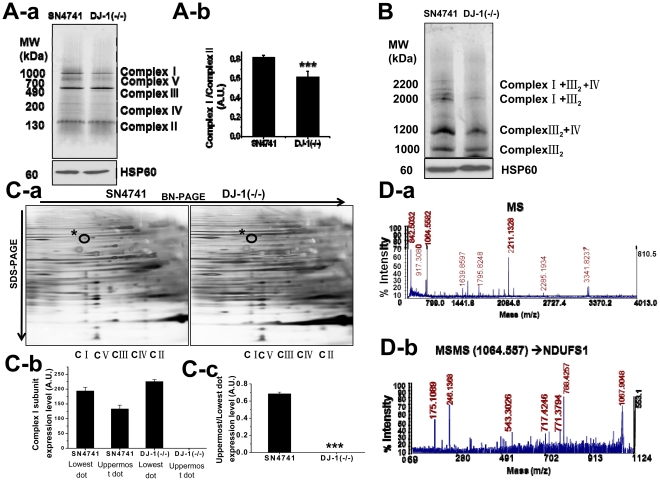
Identification of abnormalities of respiratory complex subunits in DJ-1-deficient cells. **(A-a):** SN4741 represents dopaminergic neuronal cells and DJ-1(−/−) represents dopaminergic neuronal cells homozygous for a DJ-1 gene deletion. To compare the native state of the respiratory complexes in these cell lines, isolated mitochondria were solubilized by n-dodecyl-β-d-maltoside (DDM) and resolved by using blue native-polyacrylamide gel electrophoresis (BN-PAGE). After transferring to a nitrocellulose membrane, the blot was incubated with monoclonal antibodies against five mitochondrial complex subunits using MS601 antibody cocktail (MitoSciences, Eugene, OR). The molecular weight of mitochondrial complex I was about 1000 kDa. Normalization of isolated mitochondria was achieved based on HSP60 protein expression level. The result shown is representative of three independent experiments. **(A-b):** Protein expression levels were analyzed quantitatively by densitometry. Error bars represent the mean ± SD. Significant difference compared to SN4741 cells: ***, p<0.001. **(B):** Isolated mitochondria solubilized by digitonin were analyzed by BN-PAGE for supercomplex detection. Supercomplex was detected by using MS601 monoclonal antibody cocktail as described above. I+III_2_+IV: supercomplex formed of complex I, dimeric complex III, and complex IV. I+III_2_: supercomplex formed of complex I and dimeric complex III. III_2_+IV: supercomplex formed of dimeric complex III and complex IV. III_2_: supercomplex formed of dimeric complex III **(C-a):** 2D gel analysis. BN-PAGE was performed with mitochondria isolated from SN4741 cells or DJ-1 null cells to obtain the first dimension of resolved native complexes. By using BN-PAGE gels strips, further separation in the second dimension was performed by SDS-PAGE. In DJ- null cells, in the complex I lane of the 2D gel, the uppermost dot is missing; compare the circles marked by asterisks in the two panels. **(C-b,c):** Quantification of complex I ‘dot’ levels. Two dots present in SN4741 cells were compared to the dots of DJ-1 null cells in the same position. The upper dot levels in each cell line were normalized to the lowest dot in the complex I lane of the respective cell lines and the value obtained for the DJ-1 null cells was compared to that of the SN4741 cells; the results are derived from three independent experiments. **(D-a, b):** The spot on the gel was analyzed by mass spectroscopy and determined to be the complex I subunit NDUFS1.

Because translation of the mitochondrial complex subunits was not affected, we next focused on the assembly pathway for complex I [13]. We performed BN-PAGE to detect complex I, which has a high molecular weight, to investigate whether the complex is able to assemble in DJ-1 null cells. We observed decreased complex I formation in DJ-1 null cells (Figure 1A), consistent with our previous observations of decreased complex I activity [14] (Figure S2). In the random diffusion model of electron transfer, mitochondrial complexes are postulated to exist as a supercomplex in the respiratory chain [13], [15]. More than 80% of complex I is bound to complex III or IV (or both) under physiologic conditions in which respiratory chain activity is maintained [15]. By using digitonin as a detergent and BN-PAGE, we could detect such supercomplexes (Figure 1B) and found that mitochondrial supercomplex formation was decreased in DJ-1 null cells. We postulated that the aberrant formation of mitochondrial supercomplex and native complex I was due to a defect in assembly of complex I [16]. Mitochondrial complex I assembly occurs in a step-by-step manner [13]. After assembly of mitochondrion-encoded respiratory complex subunits, the nucleus-encoded respiratory complex subunits are then assembled via assembly factors such as B17.2L [17] and Ind1 [18]. Because DJ-1 localizes to both the cytosol and nucleus, we expect that DJ-1 is associated with the nucleus-originating mitochondrial subunits, which are mostly assembled during the last steps of the mitochondrial assembly pathway [13]. To determine whether the mitochondrial complex defect observed in DJ-1 null cells is related to mitochondrial complex I formation, we performed two-dimensional (2D) gel analysis of the mitochondrial native complexes. We found that expression of the uppermost dot protein is lost in DJ-1 null cells (Figure 1C-a). Furthermore, normalization of the proteins loaded in the two gels (DJ-1 null vs. SN4741) revealed significant reductions in expression of complex I proteins in DJ-1 null cells (Figure 1C-b, c). Finally, we used MS/MS analysis to identify the missing dot as NDUFS1 which is a nucleus-encoded mitochondrial subunit of complex I (Figure 1D-a, b).

### Complex I deficiency causes mitochondrial dysfunction

Oxidative damage to complex I subunits results in increased levels of protein carbonylation and a reduced electron transfer rate, suggesting that defective assembly of complex I could contribute to PD pathogenesis by altering mitochondrial function [19]. Therefore, we next investigated functional changes in mitochondria in DJ-1 null dopaminergic neuronal cells.

**Figure 2:**
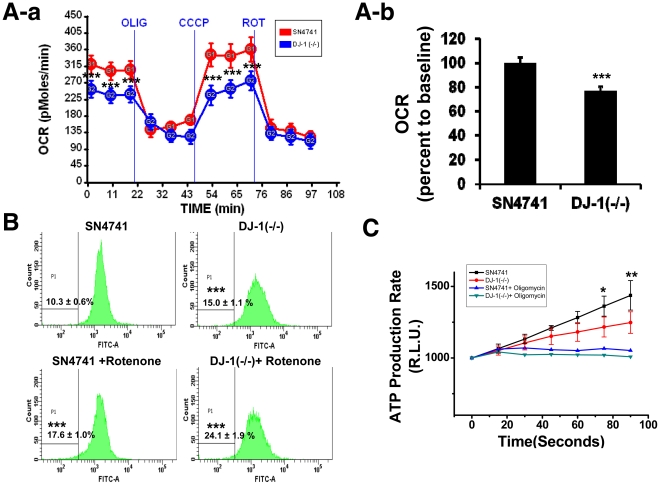
DJ-1 gene deletion decreases mitochondrial respiratory chain function in dopaminergic neuronal cells. **(A-a, b):** The O_2_ consumption rate (OCR) was measured in SN4741 cells and DJ-1 null cells in at least three independent experiments by using an XF analyzer. For validation of the measured O_2_ consumption rate, we used the 2 µg/ml oligomycin, 5 µM CCCP, and 1 µM rotenone sequentially. Each time point represents the mean (±SD), and compensated OCR data without background levels are shown in (b) with bar graphs. ***, p<0.001 **(B):** Mitochondrial membrane potential was investigated by rhodamine 123 staining and quantified by FACS analysis. ***, p<0.001 **(C):** The cell pellet was solubilized by digitonin and the mitochondrial ATP production rate was measured by a luminometer. To confirm that the calculated luminescence values represented ATP content, oligomycin was used to inhibit ATP production. *, p<0.05; **, p<0.01.

Basal oxygen consumption was significantly lower in DJ-1 null cells compared to SN4741 cells, whereas no difference in oxygen consumption was observed following treatment of the cells with oligomycin (an inhibitor of ATP synthase), which serves as a negative control (Figure 2A-a). Oxygen consumption was also lower in DJ-1 null cells compared to SN4741 cells after treatment with CCCP, which uncouples proton pumping from ATP synthesis and represents a positive control for oxygen consumption (Figure 2A-a). To normalize the level of oxygen consumption, basal oxygen consumption was compared in cells treated with rotenone (which disrupts electron transfer from complex I to ubiquinone) to give a non-mitochondrial oxygen consumption value: overall, basal respiration was about 23% lower in DJ-1 null cells compared to SN4741 cells (Figure 2A-b).

Furthermore, DJ-1 null cells exhibited a slightly increased accumulation of rhodamine 123 dye in the inner mitochondrial membrane (as measured by low-intensity fluorescence), indicating decreased mitochondrial membrane potential (Figure 2B). Thus, the observed reductions in both oxygen consumption rate and mitochondrial membrane potential suggest disruption of ATP production in the mitochondrial respiratory chain. Indeed, the rate of ATP production in DJ-1 null cells was significantly lower than in SN4741 cells, as measured by comparing with the basal level induced by treatment with oligomycin (Figure 2C).

### Structural abnormalities of mitochondria in DJ-1 null cells

**Figure 3:**
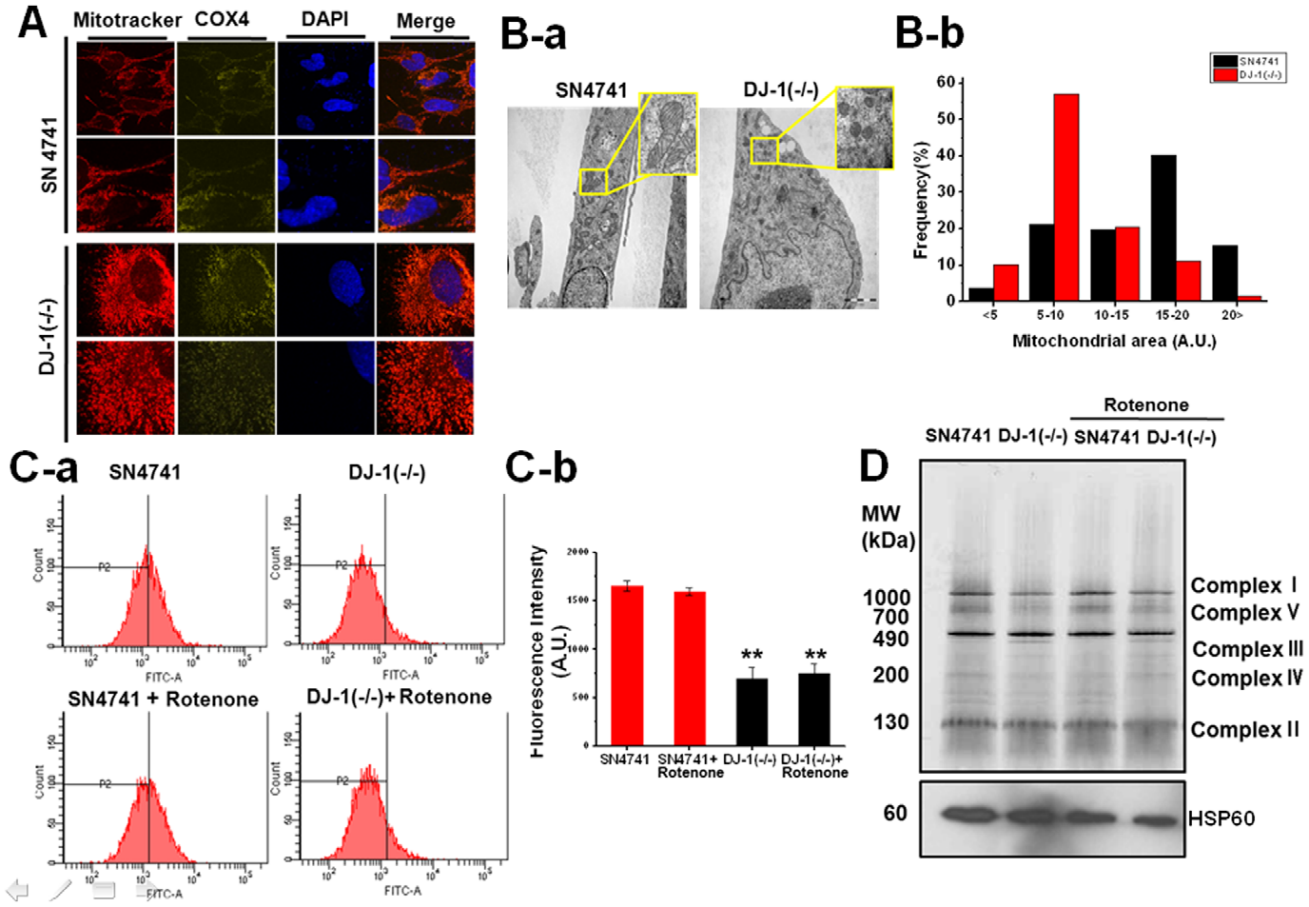
Fissional/fragmental changes of mitochondrial structure in DJ-1 null cells. **(A):** To identify mitochondrial morphologic changes, SN4741 cells and DJ-1 null cells were stained with MitoTracker red and co-stained with antibodies to COX4, which is a subunit of complex IV. The nucleus was identified by DAPI staining. The cells were sequentially observed by confocal microscopy at a magnification of ×400. **(B-a):** Cells were fixed, cryosectioned, and observed by transmission electron microscopy (TEM). DJ-1 null cells exhibit smaller mitochondria, which is apparent at a higher magnification (inset, ×50,000). The original magnification was ×15,000. **(B-b):** Mitochondrial area was analyzed by Image J software. A minimum of 20 TEM slides was evaluated to characterize mitochondrial dynamics. We divided the section equally between the largest area and the smallest area of mitochondria and measured the number of mitochondria in that section. Compared to SN4741 cells, most of the DJ-1 null cells had smaller mitochondria. **(C-a, b):** Mitochondrial mass was evaluated by MitoTracker green staining and quantified by FACS analysis. DJ-1 null cells showed a left shift in the curve, indicating reduced mitochondrial mass. Significant differences were consistently observed in three independent experiments. ***, p<0.001 **(D):** Rotenone (10 nM), an inhibitor of complex I, did not induce changes in mitochondrial complex native protein as assessed by BN-PAGE analysis. HSP60 was used as a loading control. *** p<0.001.

**Figure 4:**
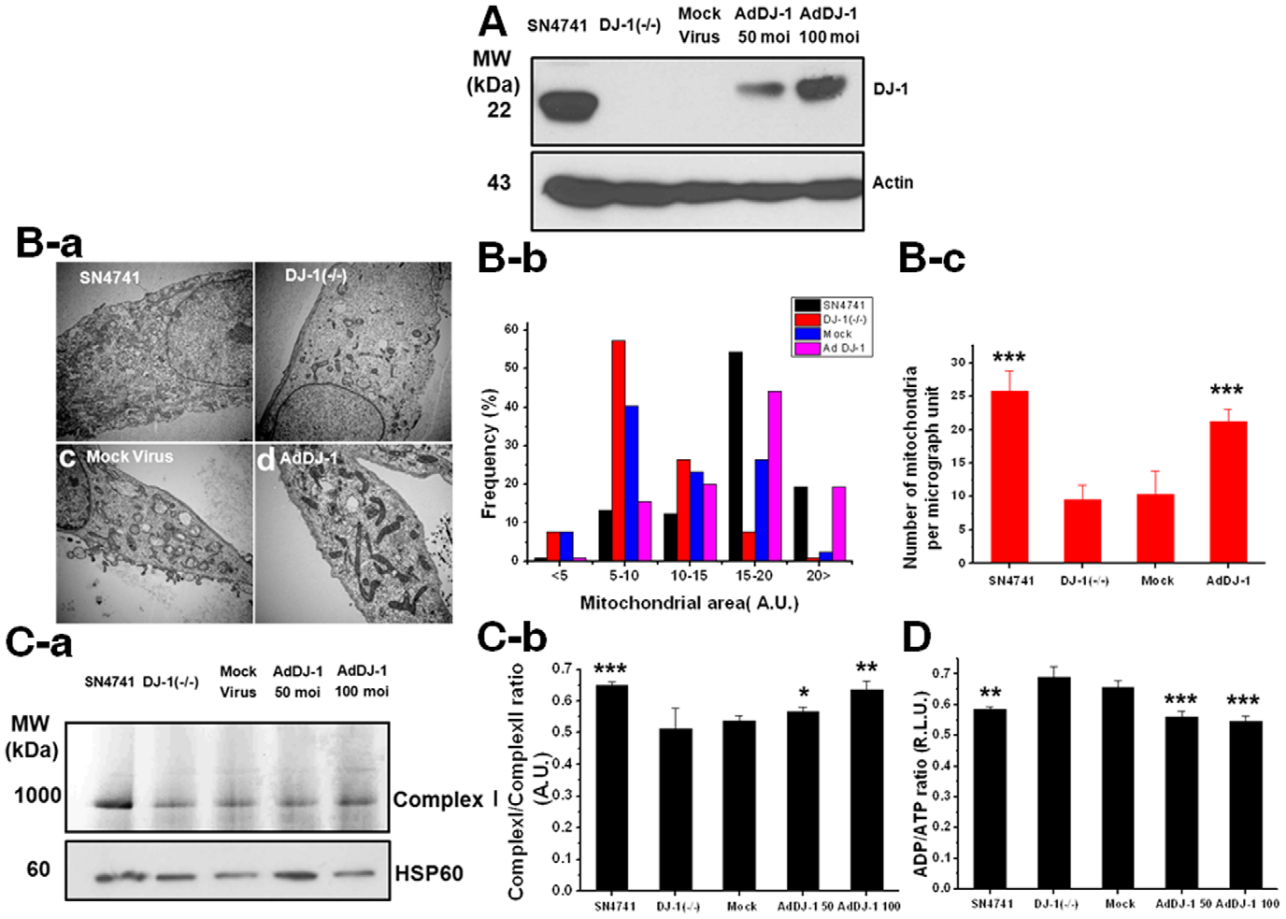
Recovery of mitochondrial assembly by overexpression of DJ-1 reverses mitochondrial defects. **(A):** Forty-eight hours after transfection of AdDJ-1, overexpression of DJ-1 protein was confirmed by western blotting. **(B-a, b, c):** Mitochondrial structure was evaluated by TEM and quantified by mitochondrial area and number. **(C-a):** Overexpression of AdDJ-1 rescued mitochondrial complex I structure, as revealed by BN-PAGE. **(C-b):** Statistical analysis of protein expression levels showed a significant recovery by AdDJ-1 compared to transfection with mock virus. **(D):** The ADP/ATP ratio was measured by fluorometry using an ApoSensor kit. *, p<0.05; **, p<0.01; *** p<0.001.

Mitochondrial defects may be involved in the early stages of PD pathogenesis [20]. In addition to mitochondrial functional defects, disruption of the fission/fusion machinery is considered an important factor in PD pathogenesis, leading to decreased mitochondrial energy production, increased oxidative stress, and impaired calcium homeostasis [21]. Therefore, we examined mitochondrial morphology in DJ-1 null cells by confocal microscopy. We found the mitochondria to be smaller in DJ-1 null cells than in SN4741 cells (Figure 3A). Furthermore, electron microscopy revealed that most DJ-1 null cells had smaller mitochondrial areas than SN4741 cells (Figure 3B-a,b). However, obvious defects of the mitochondrial matrix or mitochondrial swelling were not observed. In addition to the smaller mitochondrial size, the numbers of mitochondria were reduced in DJ-1 null cells although DJ-1 null cells were bigger than SN4741 cells (Figure 4B-a,c). We also checked the viable mitochondrial mass by staining cells with MitoTracker green dye and analyzing them by FACS. The viable mitochondrial mass was lower in DJ-1 null cells compared to SN4741 cells, indicating that both mitochondrial size and number were reduced in the cells (Figure 3C).

Rotenone, an inhibitor of mitochondrial complex I that causes PD symptoms by producing ROS, sequentially induces fissional morphologic changes [19], [22]. Therefore, we treated cells with rotenone to investigate potential involvement of the complex I assembly pathway in the mitochondrial defect of DJ-1 null cells. We determined the concentration of rotenone that was not cytotoxic to DJ-1 null and SN4741 cells (Figure S3) and subsequently treated these cells with10 nM rotenone for 24 h to measure the change in mitochondrial membrane potential and identify disruption of mitochondrial function (Figure 2B). However, the rotenone-treated cells did not exhibit decreased mitochondrial mass or decreased mitochondrial complex I assembly (Figures 3C and 3D). Unlike the mechanism of rotenone-induced dopaminergic neuronal cell death, our results support the hypothesis that DJ-1 dysfunction causes mitochondrial fragmentation through a defect in assembly of complex I.

### Overexpression of wild-type DJ-1 abrogates the complex I assembly defect

As demonstrated above, DJ-1 deficiency results in dopaminergic neuronal cells having smaller mitochondria (Figure 3A), consistent with findings in DJ-1 null mouse embryonic fibroblasts [12]. To demonstrate that the mitochondrial defects are indeed caused by DJ-1 deficiency in DJ-1 null cells, we generated an adenovirus encoding wild-type DJ-1 (AdDJ-1) to rescue DJ-1 function in mitochondrial complex assembly. In contrast to transfection of the mock virus, AdDJ-1 transfection resulted in increased DJ-1 protein expression in DJ-1 null cells (Figure 4A). AdDJ-1 transfection rescued the mitochondrial fragmention and fission defects of the null cells (Figure 4B). We also observed that the native complex I expression level was increased after AdDJ-1 transfection, based on BN-PAGE analysis (Figures 4C-a, b). Finally, we also measured the ATP/ADP ratio in DJ-1 null and AdDJ-1-transfected cells to determine mitochondrial functional changes. Overexpression of DJ-1 in DJ-1 null cells restored the ATP/ADP ratio to the level seen in SN4741 cells (Figure 4D).

## Discussion

One of the least understood mechanisms of PD pathogenesis is how a genetic defect induces dopaminergic neuronal cell death [20]. Ubiquitin-proteasomal system (UPS) defects and mitochondrial dysfunction have been implicated in neuronal cell death resulting from genetic dysfunction in PD [23]. The UPS may play a role in detoxification and targeting of dysfunctional proteins for degradation. On the other hand, because mitochondria are the primary target of PD-related toxins and supply the energy to neuronal cells, mitochondria are likely to be the first organelle to mediate neuronal cell death. In a previous study, we reported that mitochondrial complex I activity of DJ-1 null cells is specifically reduced [14]. In the present study, we determined that DJ-1 has a role in the assembly of complex I and that DJ-1 mutation causes both mitochondrial structural and functional defects, which may be the basis for susceptibility to dopaminergic neuronal cell death.

Recently, a role(s) for DJ-1 in mitochondria was suggested. Translocation of DJ-1 to mitochondria can prevent cell death by lowering mitochondrial ROS [24]. Furthermore, DJ-1 appears to be involved in removing damaged mitochondria by activating mitophagy [11]. In addition, our data show that loss of DJ-1 induces mitochondrial fragmentation and fissional morphologic changes. These findings suggest the DJ-1 mutation induces mitochondrial defects, affecting especially the mitochondrial respiratory chain, which is the major site of ROS production in the cell. In addition to these phenotypic analyses of DJ-1 function in mitochondria, Hayashi et al. reported that DJ-1 binds to the complex I subunits ND1 and NDUFS4 in yeast two-hybrid assays [25]. Therefore, although the possibility of a direct interaction between mitochondria and DJ-1 has been suggested, it is not known how DJ-1 contributes to the stability of complex I. Our BN-PAGE analysis revealed that DJ-1 is involved in complex I formation. We additionally used 2D analysis to show that aberrant formation of complex I in DJ-1 null cells occurs without NDUFS1, which is localized in the matrix arm and is the highly conserved core subunit of the Fe-S center. Mutation of *ndufs1* is associated with genetic diseases such as leukoencephalopathy and Leigh syndrome [23], [26]. However, the relevance of *ndufs1* mutation to PD and how it affects PD pathogenesis is not clear.

A cohort study of individual mitochondrial subunits could explain only 40% of patients with mitochondrion-related diseases [23]. Therefore, assembly or stability factors may also be responsible for the development of mitochondrial dysfunction-related diseases [27]. It is very interesting that we identified DJ-1 as an assembly factor for complex I. To date, six assembly factors are known to be involved in complex I assembly [28]. However, none of these assembly factors has been implicated in PD pathogenesis. In principle, assembly-factor defects could disrupt complex I activity and subsequently induce mitochondrial fragmentation, the same processes that are heavily implicated in PD pathogenesis. Consistent with this model, defects in mitochondrial function and structure in DJ-1 null cells result from defects in assembly of complex I and could be considered as a potential causative factor in PD development.

There is some evidence that DJ-1 regulates dopaminergic neuronal pacemaking [29]. In DJ-1 null cells, down-regulated expression of uncoupling proteins (UCP4 and UCP5) decreases the mitochondrial membrane potential and generates mitochondrial oxidative stress. It therefore appears that the most important function of DJ-1 is the maintenance of mitochondrial membrane potential. According to our results, DJ-1 null cells have a reduced rate of O_2_ consumption and an abnormal mitochondrial membrane potential. This shows that DJ-1, by means of its assembly factor function, has an additional mechanism for maintaining mitochondrial membrane potential.

In a previous study we found that DJ-1 null cells generate higher levels of mitochondrial superoxide compared to SN4741 cells. In the present study we focused on DJ-1 function in complex I assembly as one of the factors affecting mitochondrial ROS generation. Along with the pacemaking function of DJ-1 by regulation of UCP channels, our results suggest that DJ-1 also maintains mitochondrial membrane potential by sustaining complex I formation. We propose that neuronal cell death due to DJ-1 mutation is caused by aberrant assembly or maintenance of complex I and the consequent failures of mitochondrial function and structure.

## Materials and Methods

### Cell lines and culture conditions

Establishment of the dopaminergic neuronal cell line SN4741 and the DJ-1 null dopaminergic neuronal cell line was described previously [30]; both cell lines were kindly provided by Dr. Son (Ewha Womans University in Korea). Briefly, SN4741 DJ-1+ and DJ-1 null cells were established from the substantia nigra region of E13.5 “wild-type” and double DJ-1 knockout mouse embryos, respectively. The cells were characterized for expression of the general neuronal markers TuJ1, NeuN and tyrosine hydroxylase by western blot analysis. Cells were grown in RF medium containing Dulbecco's modified Eagle's medium (DMEM, Gibco) supplemented with 10% (v/v) heat-inactivated fetal bovine serum (Gibco), 1% glucose, penicillin (100 units/ml)–streptomycin (100 mg/ml), and l-glutamine (2 mM) at 33°C with 5% CO_2_, as described previously [30].

### Reagents

Digitonin was purchased from Sigma (Sigma-Aldrich, St. Louis, MO, USA) and dissolved in dimethyl sulfoxide (DMSO). MitoTracker red, green, and rhodamine 123 were purchased from Molecular Probes (USA) and dissolved in distilled water. Oligomycin and rotenone were dissolved in ethanol. Adenosine pentaphosphate, malate, pyruvate, succinate, and luciferin were dissolved in distilled water. The anti-DJ-1 rabbit polyclonal antibody was purchased from Novus Biological (Novus, Littleton, CO, USA). The anti-COX4 mouse monoclonal antibody and anti-ß-actin rabbit polyclonal antibody were purchased from Santa Cruz Biotechnology (Santa Cruz, CA, USA). For detection of mitochondrial complexes in BN-PAGE, we used the anti-MS601 antibody cocktail (MitoSciences, Eugene, OR).

### Isolation of mitochondria

Isolation of mitochondria was performed as described [31]. Cells were suspended in buffer A (250 mM sucrose, 2 mM HEPES, pH 7.4, and 0.1 mM EGTA) and centrifuged at 320× *g* for 10 min. Cell pellets were homogenized in buffer A with a glass-teflon homogenizer. The homogenate was centrifuged at 570× *g* for 10 min and the supernatant was retained. For crude mitochondria preparation, the supernatant was centrifuged at 14,000× *g* for 10 min. The pellet was resuspended in buffer B (25 mM potassium phosphate, pH 7.2 and 5 mM MgCl_2_) and centrifuged at 15,000× *g* for 10 min. The mitochondrial pellet was used or stored at −70°C for BN-PAGE. The mitochondrial preparation was validated by using mitochondrion-specific antibody (anti-COX4) and western blotting. To rule out contamination by nuclear or cytosolic proteins, the isolated mitochondria were evaluated with Lamin B antibody as a nuclear protein marker and copper-zinc superoxide dismutase and tubulin antibodies as cytosolic markers (Figure S4).

### BN-PAGE analysis

One-dimensional and 2D BN-PAGE analyses were performed with isolated mitochondria that were lysed with *n*-dodecyl-β-d-maltoside using the Native PAGE TM Novex® Bis-Tris Gel system (Invitrogen, USA) according to the manufacturer's instructions. Briefly, 30 µg of isolated mitochondria were solubilized using sodium dodecyl maltoside. Digitonin was used in the lysis buffer for detection of the mitochondrial supercomplex. The suspensions were centrifuged at 20,000× *g* for 10 min at 4°C. The resulting supernatants were loaded onto a native polyacrylamide Novex 3–12% Bis-Tris Gel, and electrophoresis (PAGE) was performed, and the proteins were transferred to a polyvinylidene fluoride (PVDF) membrane. After fixing with 8% acetic acid, the membrane was blocked with 5% skim milk in TBS-T (10 mM Tris-HCl, pH 7.6, 150 mM NaCl and 0.1% Tween 20) for 1 h. Anti-OxPhos Complex Kit (Invitrogen) antibody was used. The proteins were detected using Western Breeze® (Invitrogen) Chromogenic Western Blot Immunodetection Kit. For 2D analysis, sodium dodecyl sulfate (SDS)-PAGE was performed and the gel was stained with silver nitrate.

### In-gel digestion

Stained proteins were excised from the gels and destained with a solution containing 30 mM potassium ferricyanide and 100 mM sodium thiosulfate. The gel slices were rinsed several times with distilled water to stop the reaction. The gel slices were dried and incubated in a solution containing 10 mM dithiothreitol and 100 mM ammonium bicarbonate at 56°C to reduce protein disulfide bonds, followed by incubation in 100 mM iodoacetamide to alkylate cysteines. The gel slices were then washed with 2–3 volumes of distilled water and dried using a speed vacuum concentrator. After swelling the gels with 30 µl of 50 mM ammonium bicarbonate, proteins were digested with 7–8 µl trypsin (0.1 µg/µl) at 37°C for 12–16 h. The digested peptides were then recovered using 2 extractions and the peptide extracts were pooled. Pooled extracts were lyophilized in a vacuum centrifuge and dissolved in 0.5% TFA solution before MS or MS/MS analysis.

### Protein identification by LC-MS and MS/MS

To improve the ionization efficiency of MALDI TOF/MS, sample peptides were desalted using a Zip-Tip C18 (Millipore, USA). Peptides were eluted onto MALDI target plates using matrix solution (10 mg/ml α-cyano-4-hydroxycinnamic acid dissolved in a solution consisting of 50% acetonitrile and 0.5% TFA). All mass spectra were acquired in reflection mode using a 4700 proteomic analyzer (Applied Biosystems, Framingham, MA, USA). When the PMF results were not satisfactory, MS/MS search results were used to support the PMF results. Protein identification and quantification were performed using Mascot version 2.2 software (Matrix Science Inc., Boston, MA).

### Measurement of mitochondrial oxygen consumption

Cellular oxygen consumption was measured using a Seahorse Bioscience XF24 analyzer (Seahorse Bioscience Inc., USA) in 24-well plates at 37°C, with correction for positional temperature variations adjusted from 4 empty wells evenly distributed within the plate [32]. SN4741 and DJ-1 null cells were seeded at 25,000 cells per well during 18 hours prior to the analysis and each experimental condition was performed on 7 biological replicates. Before each measurement, the cells were washed and 590 µl of non-buffered medium (sodium bicarbonate-free DMEM, pH 7.4) was added to each well. After a 15-min equilibration period, three successive 2-min measurements were performed at 3-min intervals with inter-measurement mixing to homogenize the oxygen concentration in the medium, and each condition was measured in independent wells. Concentrated compounds (10×) were injected into each well by using the internal injectors of the cartridge and three successive 2-min measurements were performed at 3-min intervals with inter-measurement mixing.

### Measurement of mitochondrial membrane potential

Mitochondrial membrane potential was evaluated using rhodamine 123 dye, which is a positively molecule that is sensitive to the proton gradient and can accumulate in energized mitochondria [33]. DJ-1 null cells and SN4741 cells were grown in 6-well plates for 24 h and washed with PBS 3 times. Cells were stained with rhodamine 123 for 15 min at 37°C in an incubator. Cells were trypsinized, centrifuged at 800× *g* at room temperature (RT), and resuspended in PBS (pH 7.4). Samples were analyzed on a FACScan (BD Biosciences, Bedford, MA, USA) and data analysis was performed with BD FACSDiva software (BD Biosciences, Bedford, MA, USA). To verify the membrane potential measurements, we used high doses of the mitochondrial complex inhibitors rotenone, oligomycin and CCCP (Figure S5).

### Analysis of mitochondrial ATP synthesis rate

To measure mitochondrial ATP synthesis rate, cells were harvested by trypsinization, centrifuged at 800× *g* at RT, and the cell pellet was washed with glucose and serum-free medium [34]. Cells were incubated in buffer A [150 mM KCl, 25 mM Tris-HCl, 2 mM EDTA, 0.1% BSA, 10 mM potassium phosphate, and 0.1 mM MgCl_2_ (pH 7.4)] for 1 min at RT to permeabilize the cell membrane. The cell pellet was resuspended in buffer A and diadenosine pentaphosphate, malate, pyruvate, and succinate were added to the cell solution. Luminometer measurements of the cell pellet and solution mixture were made immediately after addition of ADP and buffer B (0.5 M Tris-acetate, pH 7.75, 0.8 mM luciferin, and 20 µg/ml luciferase). Cells treated with oligomycin were used as the control. Oligomycin, an ATP synthase inhibitor, disrupts electron transfer in the mitochondrial respiratory chain, blocking ATP production.

### Adenovirus-mediated overexpression of DJ-1

DJ-1 was overexpressed in DJ-1 null cells by using Ad DJ-1, according to our previously reported methods [14]. The protein level of DJ-1 resulting from Ad DJ-1 transfection was evaluated by western blotting.

### ATP/ADP ratio

An ApoSensor ADP/ATP Ratio Assay Kit was used to measure the ADP/ATP ratio (BioVision, Mountain View, CA, USA). Briefly, cells were grown in 6-well plates at 33°C for 24 h. The medium was removed and 100 µl of nucleotide-releasing buffer was added for 5 min. ATP levels were assessed by the addition of 1 µl of the ATP monitoring enzyme followed by the immediate measure of ATP content by using a luminometer. After 10 min, 1 µl of ADP converting enzyme was added to measure the ADP content.

### Electron microscopic analysis

DJ-1 null and SN4741 cells were fixed in 2.5% glutaraldehyde in PBS, harvested, centrifuged, and dehydrated in a series of ethanol. The 70% ethanol step was saturated with uranyl acetate for contrast enhancement. Dehydration was completed in propylene oxide and the specimens were produced on a FCR Reichert Ultracut Ultramicrotome, mounted on pioloform-coated copper grids, and contrasted with lead citrate. Specimens were analyzed and documented with an EM 10A electron microscope. Approximately 5–10 mitochondria per slide were examined to obtain a characterization of mitochondrial dynamics by analyzing mitochondrial area. Mitochondrial area was analyzed by the Image J program with a minimum of 20 slides. The total number of analyzed mitochondrial areas was 160. We divided the section equally between the largest area and the smallest area of mitochondria and measured the number of mitochondria in that section. The number of mitochondria was counted in each cell and a total 10 cells was counted and subjected to statistical analysis.

### Immunocytochemistry

Cells were grown in 12-well plates with a cover slip for 24 h. For MitoTracker red staining, cells were incubated under culture conditions and the MitoTracker red dye was added. After 20 min, cells were fixed with 3% paraformaldehyde and permeabilized with 0.1% Triton X-100. After blocking with 1% BSA, cells were incubated with an anti-COX4 antibody (1∶250; Santa Cruz) and FITC-conjugated anti-mouse secondary antibody according to the manufacturer's instructions (Santa Cruz Biotechnology, CA, USA). Slides were cover-slipped with VECTASHIELD mounting medium and photos were taken on an Olympus™ confocal microscope at ×400 magnification.

### Preparation of cell lysates and western blot analysis

Proteins were extracted with RIPA buffer (10 mM Tris-HCl, pH 8.0, 150 mM NaCl, 1% Nonidet P-40) containing protease inhibitors (Roche, Mannheim, Germany). Protein concentrations was measured using the Bradford method [35]. Samples were resolved by 10% SDS-PAGE and transferred to Hybond ECL membranes (Amersham Pharmacia Biotech, Buckinghamshire, UK). The membrane was blocked in Tris-buffered saline containing 0.1% Tween 20 (TBS/T) with 5% nonfat skim milk for 1 h at RT and incubated with primary antibody for 1 h at RT. After 3 washes in TBS/T, the membrane was incubated with horseradish peroxidase-conjugated secondary antibody for 1 h at RT. After 3 washes in TBS/T, the membrane was visualized by enhanced chemiluminescence (Amersham Pharmacia Biotech, Buckinghamshire, UK).

### Statistical analysis

Statistical analyses were performed as recommended by an independent statistician. This included the unpaired Student's *t*-test. All values are expressed as mean ± standard deviation (SD) and p values<0.05 were considered to be statistically significant.

## Supporting Information

Figure S1 **Identification of mitochondrial respiratory chain complex subunits in SN4741 and DJ-1 null cells.** SN4741 and DJ-1 null cells were lysed in RIPA buffer and protein concentration was measured by Bradford assay. After transferring to PVDF membrane, the blots were incubated with primary antibodies to subunits of each mitochondrial complex. NDUFA9 is a subunit of complex I, SDHA is a subunit of complex II, UQCR1 is a subunit of complex III, COX4 is a subunit of complex IV, and ATP5a1 is a subunit of complex V. Beta-actin was used as a loading control. Except for COX4, no differences in expression levels between SN4741 and DJ-1 null cells were evident for any of the other mitochondrial complex subunits.
(PPTX)

Figure S2 **Reduced mitochondrial complex I activity in DJ-1 null cells.** Mitochondrial complex I activity was measured as described previously [14]. Briefly, mitochondrial extracts were disrupted by freezing and thawing 3 times in hypotonic buffer [25 mM potassium phosphate (pH 7.2), 5 mM MgCl_2_]. Complex I activity was measured by following the reduction in absorbance due to the oxidation of NADH at 340 nm for 3–5 min. The mitochondrial proteins (20–50 µg) were added in buffer containing 50 mM Tris-HCl, pH 8.1, 0.25% BSA, 0.3 mM KCN, 100 µM NADH, 50 µM CoQ, 5 µM rotenone at 37°C. Complex I-specific activity was measured with and without 5 µM rotenone for 3–5 min. Complex I activity was decreased by about 30% in DJ-1 null cells compared to SN4741 cells. This result is based on measurements from three independent experiments. *** p<0.001.
(PPTX)

Figure S3 **Viability of SN4741 cells as a function of rotenone concentration.** The viability of rotenone-treated SN4741 cells was analyzed by 3-(4,5-dimethylthiazol-2-yl)-2,5-diphenyltetrazolium bromide (MTT) assay. SN4741 cells were plated at 1×10^4^ per well in 96-well tissue culture plates and incubated at 33°C. The cultured cells were treated with rotenone. After 24 h the cells were incubated with MTT for 2 h and dissolved in DMSO and read at 570 nm using a microplate reader (VERSAmax, Molecular Devices Corp., Sunnyvale, CA). Rotenone concentrations >20 nM were found to have noticeable toxic effects on SN 4741 cells.
(PPTX)

Figure S4 **Identification of the mitochondrial fraction.** Validation of the mitochondrial fraction was performed by using organelle-specific antibody. Ant-COX4 antibody was used to detect the mitochondrial fraction, Lamin B antibody was used to detect the nuclear fraction, and CuZnSOD and tubulin were used to detect the cytosolic fraction. The isolated mitochondria expressed high levels of COX4, but did not express Lamin B, CuZnSOD or tubulin. HSP60 was used as a loading control.
(PPTX)

Figure S5 **Validation of mitochondrial membrane potential measurements by using respiratory chain inhibitors.** Mitochondrial membrane potential was detected by rhodamine 123 dye using FACS analysis. We used high doses of rotenone (200 nM) and CCCP (5 µM) as positive controls and oligomycin (2 µg/ml) as a negative control. As expected, treatment with rotenone and CCCP showed a leftward shift of the median line, which indicates that the mitochondrial membrane potential was depolarized. In contrast, treatment with oligomycin showed a rightward shift of the median line, which indicates hyperpolarization of mitochondrial membrane potential.
(PPTX)
