# Supplementary figures and images for: DJ-1 Null Dopaminergic Neuronal Cells Exhibit Defects in Mitochondrial Function and Structure: Involvement of Mitochondrial Complex I Assembly
Source: PLoS One. 2012 Mar 5;7(3):e32629. doi: 10.1371/journal.pone.0032629 (PMC3293835; doi:10.1371/journal.pone.0032629)

## Slide 1
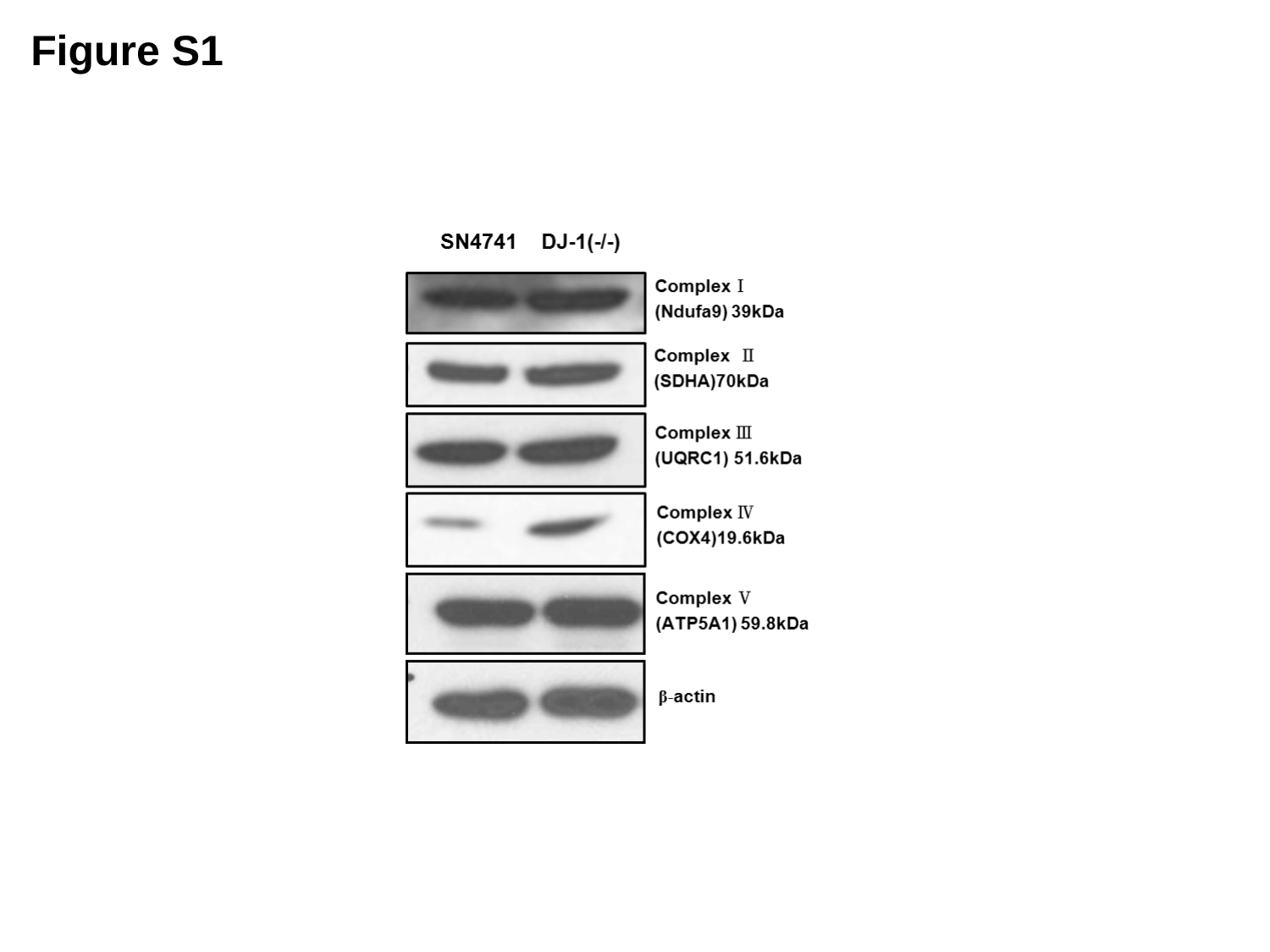

Figure S1

Supplement: Figure S1 — Identification of mitochondrial respiratory chain complex subunits in SN4741 and DJ-1 null cells. SN4741 and DJ-1 null cells were lysed in RIPA buffer and protein concentration was measured by Bradford assay. After transferring to PVDF membrane, the blots were incubated with primary antibodies to subunits of each mitochondrial complex. NDUFA9 is a subunit of complex I, SDHA is a subunit of complex II, UQCR1 is a subunit of complex III, COX4 is a subunit of complex IV, and ATP5a1 is a subunit of complex V. Beta-actin was used as a loading control. Except for COX4, no differences in expression levels between SN4741 and DJ-1 null cells were evident for any of the other mitochondrial complex subunits. (PPTX) [file pone.0032629.s001.pptx]

## Slide 1
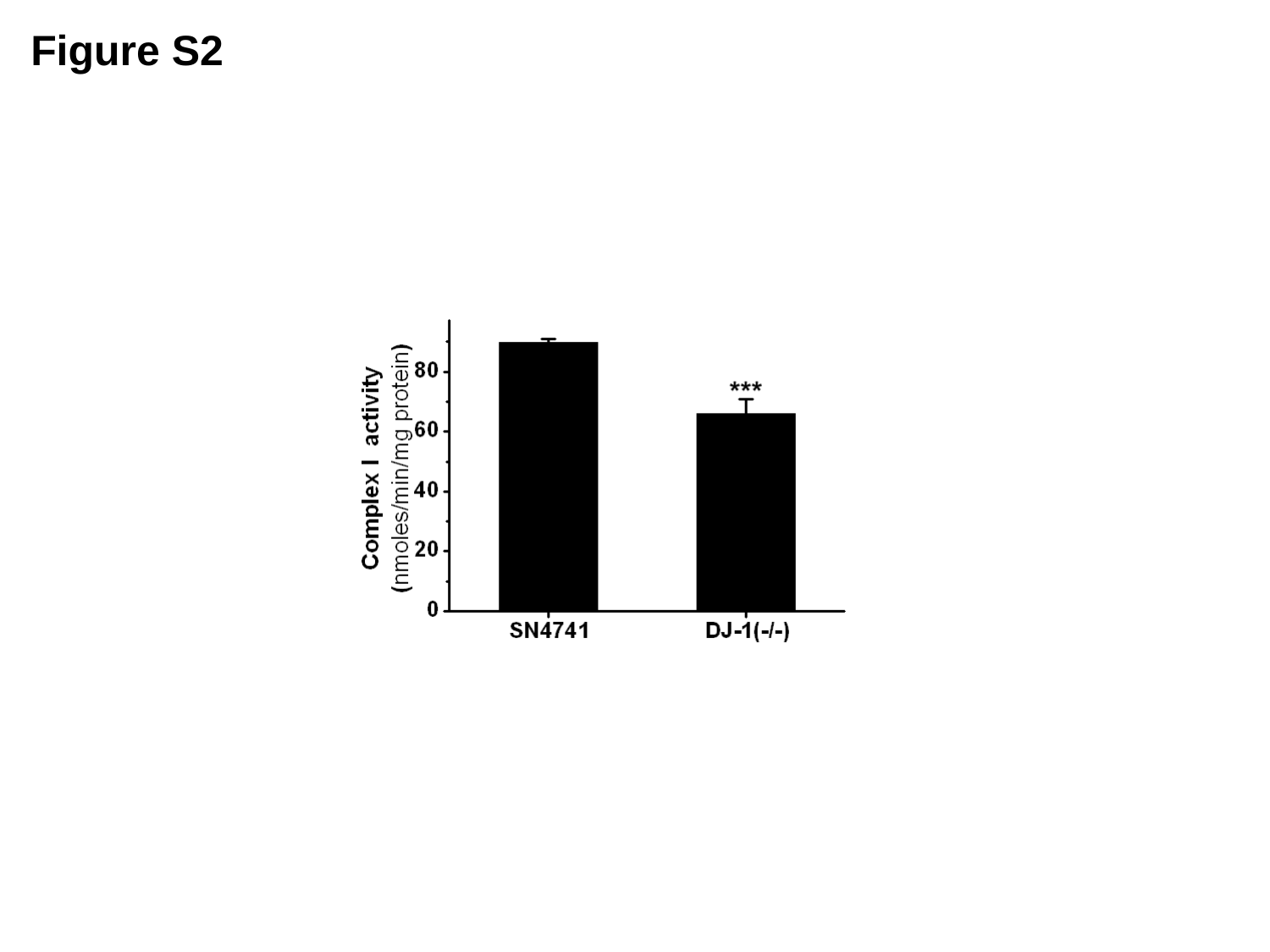

Figure S2

Supplement: Figure S2 — Reduced mitochondrial complex I activity in DJ-1 null cells. Mitochondrial complex I activity was measured as described previously [14]. Briefly, mitochondrial extracts were disrupted by freezing and thawing 3 times in hypotonic buffer [25 mM potassium phosphate (pH 7.2), 5 mM MgCl2]. Complex I activity was measured by following the reduction in absorbance due to the oxidation of NADH at 340 nm for 3–5 min. The mitochondrial proteins (20–50 µg) were added in buffer containing 50 mM Tris-HCl, pH 8.1, 0.25% BSA, 0.3 mM KCN, 100 µM NADH, 50 µM CoQ, 5 µM rotenone at 37°C. Complex I-specific activity was measured with and without 5 µM rotenone for 3–5 min. Complex I activity was decreased by about 30% in DJ-1 null cells compared to SN4741 cells. This result is based on measurements from three independent experiments. *** p<0.001. (PPTX) [file pone.0032629.s002.pptx]

## Slide 1
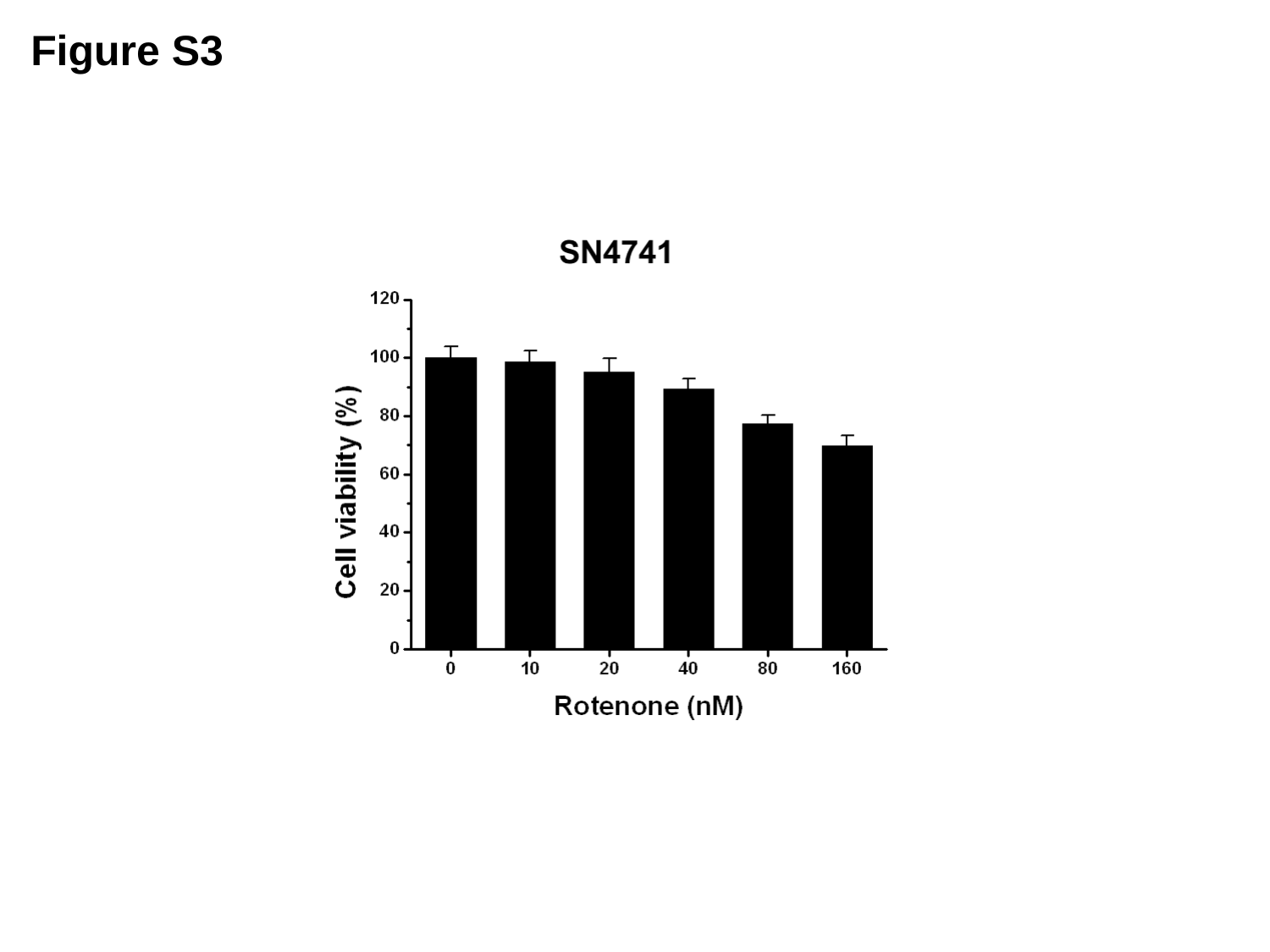

Figure S3

Supplement: Figure S3 — Viability of SN4741 cells as a function of rotenone concentration. The viability of rotenone-treated SN4741 cells was analyzed by 3-(4,5-dimethylthiazol-2-yl)-2,5-diphenyltetrazolium bromide (MTT) assay. SN4741 cells were plated at 1×10^4^ per well in 96-well tissue culture plates and incubated at 33°C. The cultured cells were treated with rotenone. After 24 h the cells were incubated with MTT for 2 h and dissolved in DMSO and read at 570 nm using a microplate reader (VERSAmax, Molecular Devices Corp., Sunnyvale, CA). Rotenone concentrations >20 nM were found to have noticeable toxic effects on SN 4741 cells. (PPTX) [file pone.0032629.s003.pptx]

## Slide 1
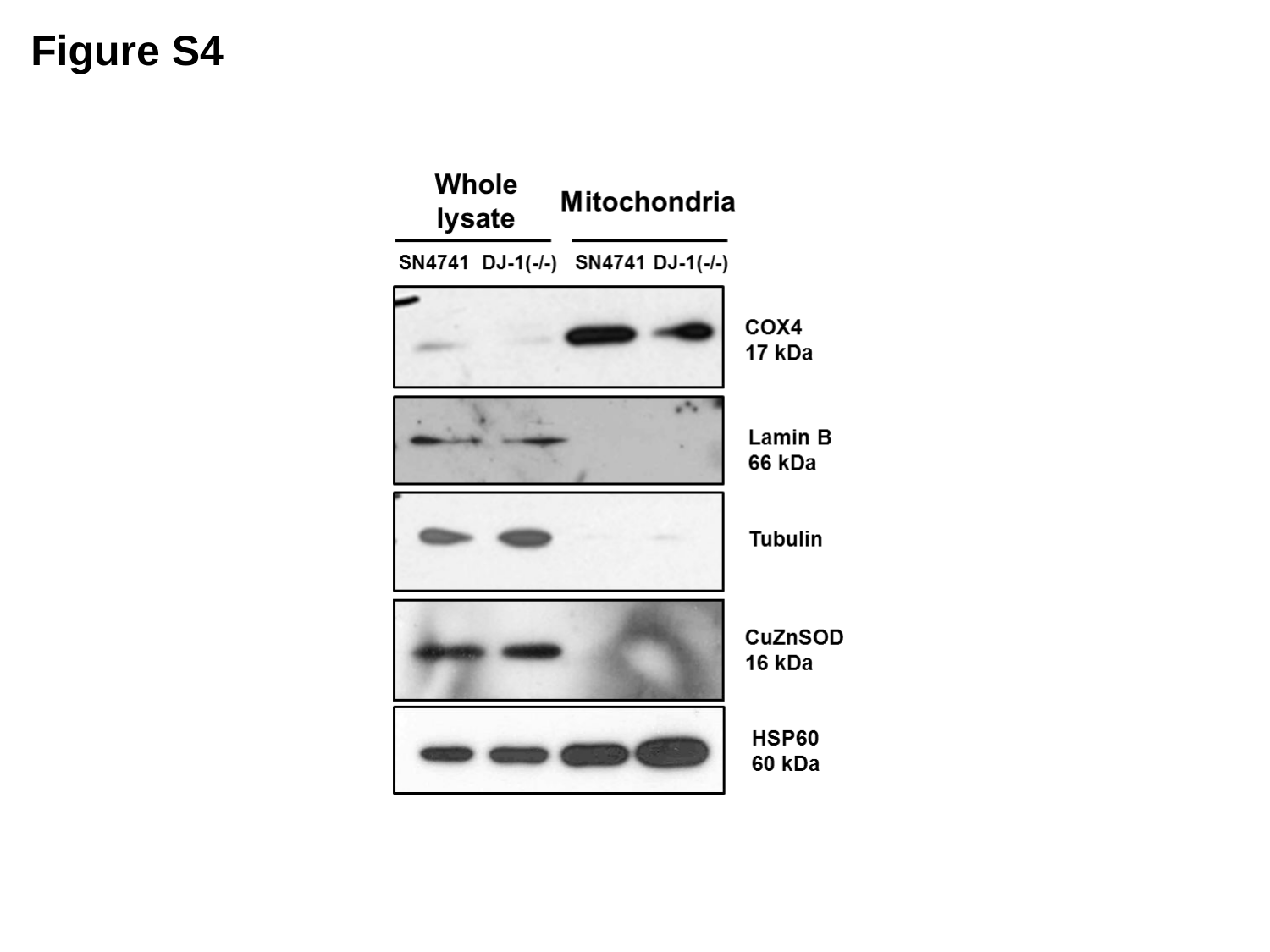

Figure S4

Supplement: Figure S4 — Identification of the mitochondrial fraction. Validation of the mitochondrial fraction was performed by using organelle-specific antibody. Ant-COX4 antibody was used to detect the mitochondrial fraction, Lamin B antibody was used to detect the nuclear fraction, and CuZnSOD and tubulin were used to detect the cytosolic fraction. The isolated mitochondria expressed high levels of COX4, but did not express Lamin B, CuZnSOD or tubulin. HSP60 was used as a loading control. (PPTX) [file pone.0032629.s004.pptx]

## Slide 1
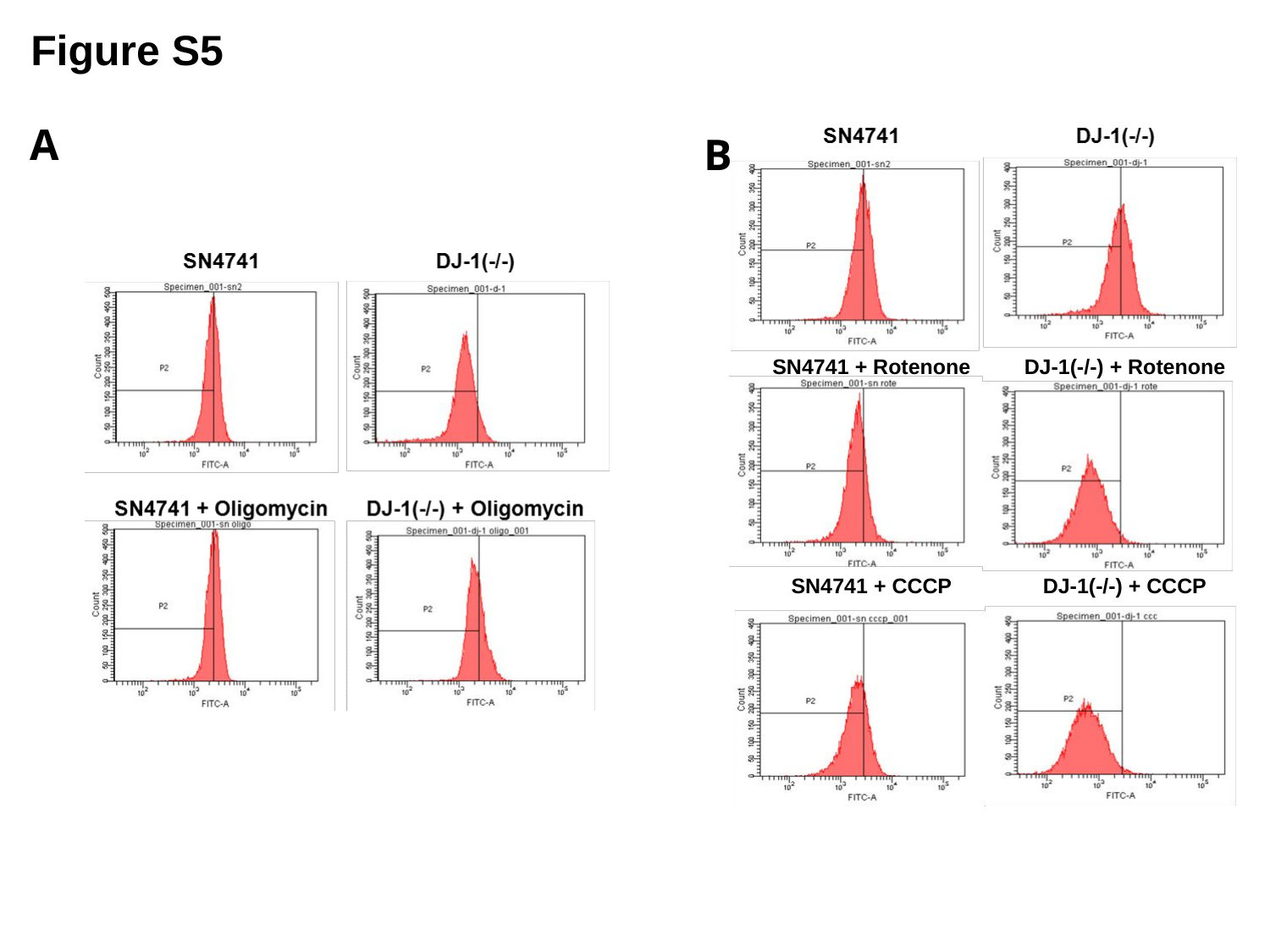

Figure S5
A
B
SN4741 + Rotenone
DJ-1(-/-) + Rotenone
SN4741 + CCCP
DJ-1(-/-) + CCCP

Supplement: Figure S5 — Validation of mitochondrial membrane potential measurements by using respiratory chain inhibitors. Mitochondrial membrane potential was detected by rhodamine 123 dye using FACS analysis. We used high doses of rotenone (200 nM) and CCCP (5 µM) as positive controls and oligomycin (2 µg/ml) as a negative control. As expected, treatment with rotenone and CCCP showed a leftward shift of the median line, which indicates that the mitochondrial membrane potential was depolarized. In contrast, treatment with oligomycin showed a rightward shift of the median line, which indicates hyperpolarization of mitochondrial membrane potential. (PPTX) [file pone.0032629.s005.pptx]
